# Supplementary material for: Liver ASK1 protects from non‐alcoholic fatty liver disease and fibrosis
Source: EMBO Mol Med. 2019 Jun 6;11(10):e10124. doi: 10.15252/emmm.201810124 (PMC6783644; doi:10.15252/emmm.201810124)
Supplement: Supplementary file 1 — Appendix [file EMMM-11-e10124-s001.pdf]

Liver ASK1 protects from non-alcoholic fatty liver disease and fibrosis

Tenagne D. Challa, Stephan Wueest, Fabrizio C. Lucchini, Mara Dedual, Salvatore Modica, Marcela Borsigova, Christian Wolfrum, Matthias Blüher, Daniel Konrad

Appendix

Appendix Figures 1-5
Appendix Tables 1-4

Appendix Figure 1

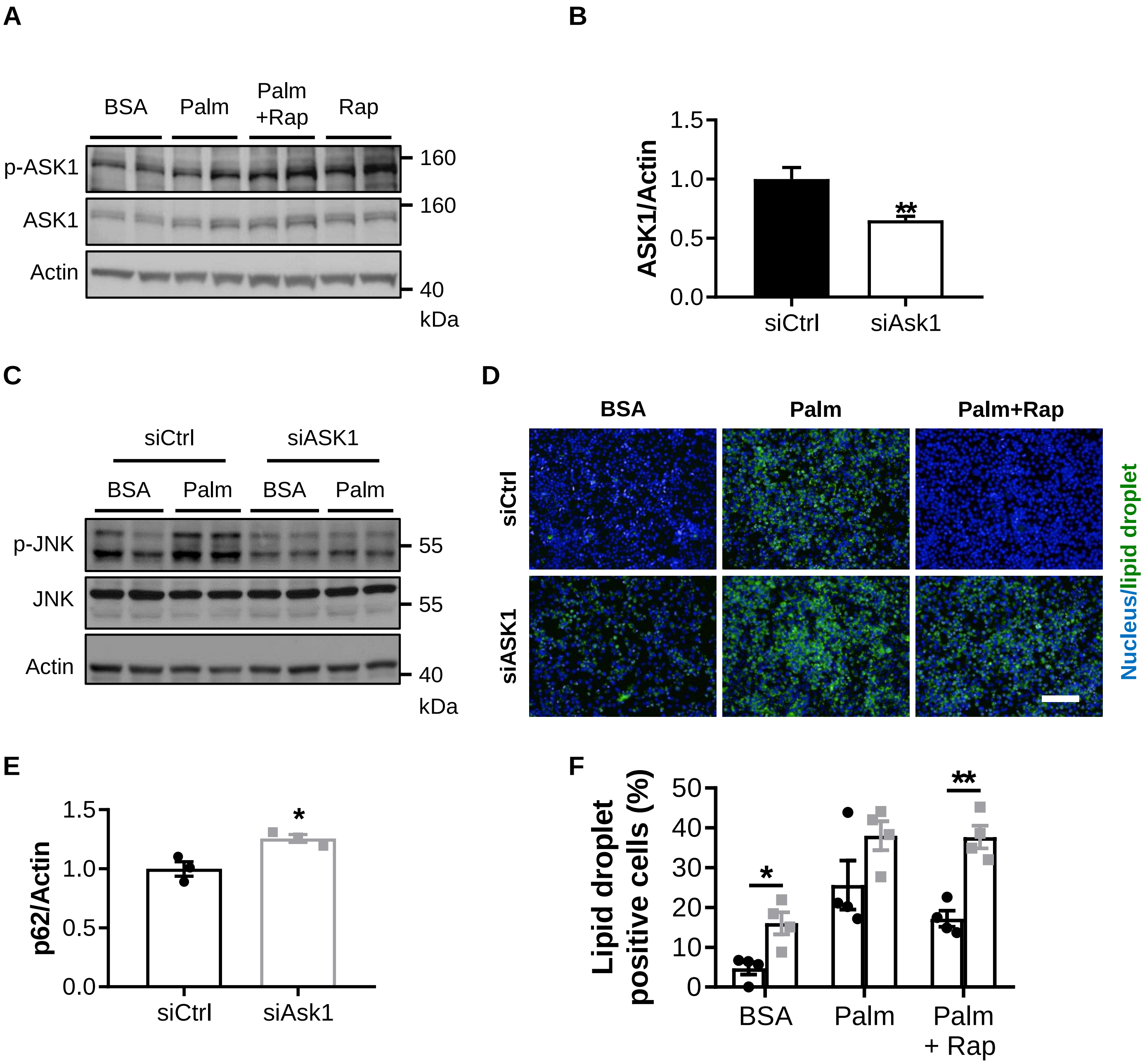

Activation of hepatic ASK1 decreases lipid storage

(**A**) p-ASK1 and ASK1 protein levels in hepatocytes treated with 0.4 mM bovine serum albumin (BSA), 0.4 mM palmitate (Palm) conjugated to BSA, 1 mM rapamycin (Rap) or Palm+Rap for 24 h (n=4). (**B**) Quantification of ASK1 protein levels in HepG2 transfected with siRNA targeting ASK1 (siASK1) or non-targeting siRNA control (siCtrl) (n=6). (**C-E**) Hepatocytes were transfected with 200 nM of siRNA targeting ASK1 (siAsk1) or non-targeting siRNA control (siCtrl) and treated with BSA, Palm, or Palm+Rap. (**C**) Representative Western blot of p-JNK in ASK1-depleted or control cells treated with BSA or Palm. (**D**) The cells were stained for lipid droplet accumulation (Bodipy 493/503, green) and nuclei (Hoechst, blue). Representative images are depicted. Scale bar represents 100 µm. (**E**) Quantification of p62 protein levels in ASK1-depleted (siASK1) or control (siCtrl) cells treated with BSA or Palm (n=3). (**F**) Colocalization of LC3 punctate with lipid droplets was quantified in hepatocytes transfected with siRNA targeting ASK1 (siASK1; grey bars) or non-targeting siRNA control (siCtrl; black bars) (n=4 biological replicates). Values are expressed as mean ± SEM. *p<0.05, **p<0.01. Statistical test used: *t*-test.

Appendix Figure 2

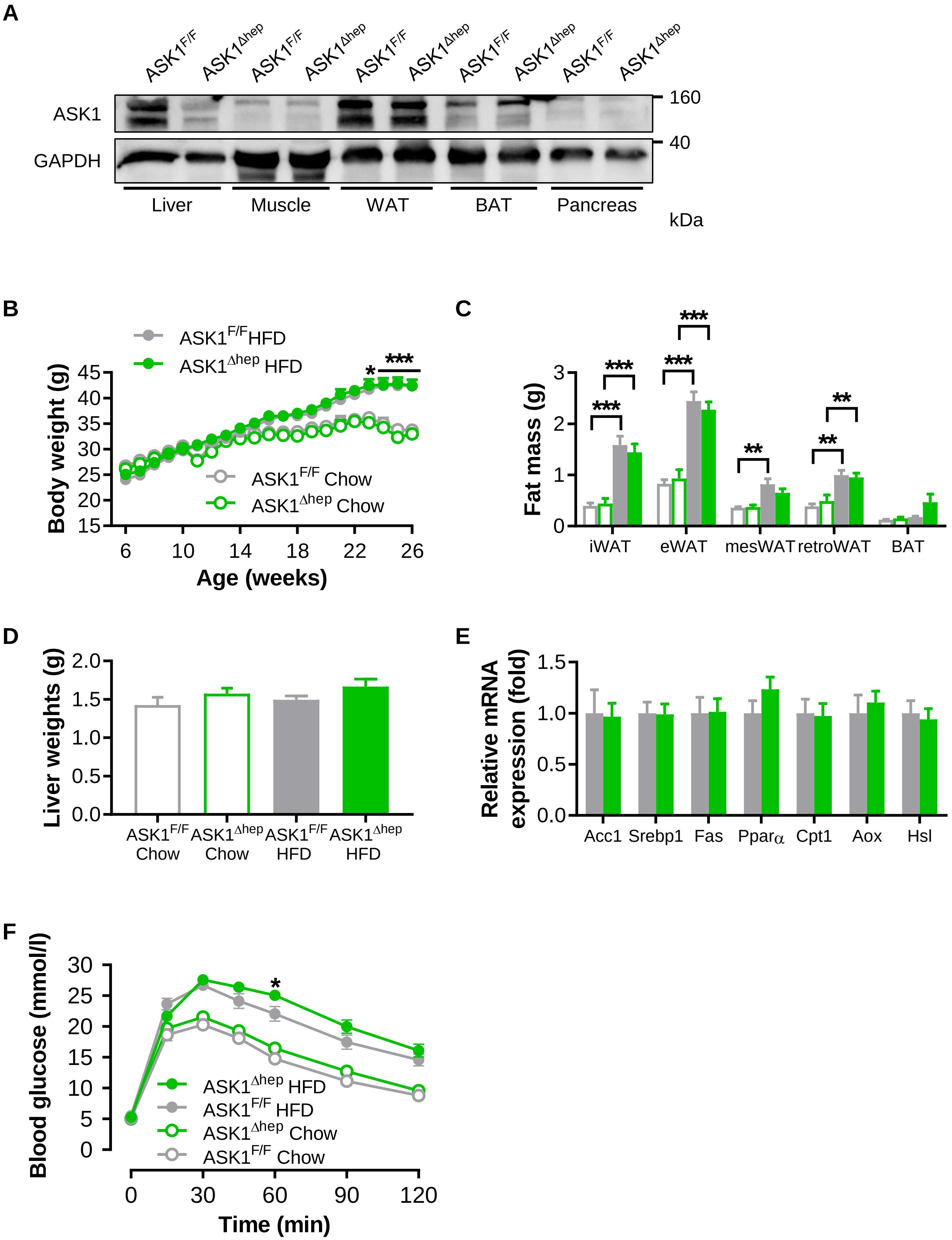

Characterization of hepatocyte-specific ASK1-knockout mice

(A) ASK1 protein levels in liver and selected tissues. (B) Body weight gain of chow- (ASK1$^{F/F}$ n=7, ○; ASK1$^{\Delta hep}$ n=6, ○) and HFD-fed (ASK1$^{F/F}$ n=21, ●; ASK1$^{\Delta hep}$ n=24, ●) mice. (C) Fat pad weight (ASK1$^{F/F}$ n=17; ASK1$^{\Delta hep}$ n=19) or chow-fed mice (ASK1$^{F/F}$ n=9; ASK1$^{\Delta hep}$ n=8) and (D) liver weight of HFD-fed (ASK1$^{F/F}$ n=21; ASK1$^{\Delta hep}$ n=24) or chow-fed mice (ASK1$^{F/F}$ n=9; ASK1$^{\Delta hep}$ n=8). (E) Relative mRNA expression of genes involved in lipolysis, lipogenesis and fatty acid oxidation in liver of mice fed a HFD for 20 weeks (ASK1$^{F/F}$ n=12; ASK1$^{\Delta hep}$ n=15 for *Srebp1, Fas, Pparα, Cpt1, Aox*; ASK1$^{F/F}$ n=6; ASK1$^{\Delta hep}$ n=7-8 for *Acc1* and *Hsl*). (F) Intraperitoneal (i.p.) glucose-tolerance test in mice fed a chow (ASK1$^{F/F}$ n=11; ASK1$^{\Delta hep}$ n=13) or HFD (ASK1$^{F/F}$ n=10; ASK1$^{\Delta hep}$ n=12. All values are expressed as mean ± SEM. *p<0.05; **p<0.01, ***p<0.001. Statistical test used: ANOVA for B, C, D, H; *t*-test for F.

Appendix Figure 3

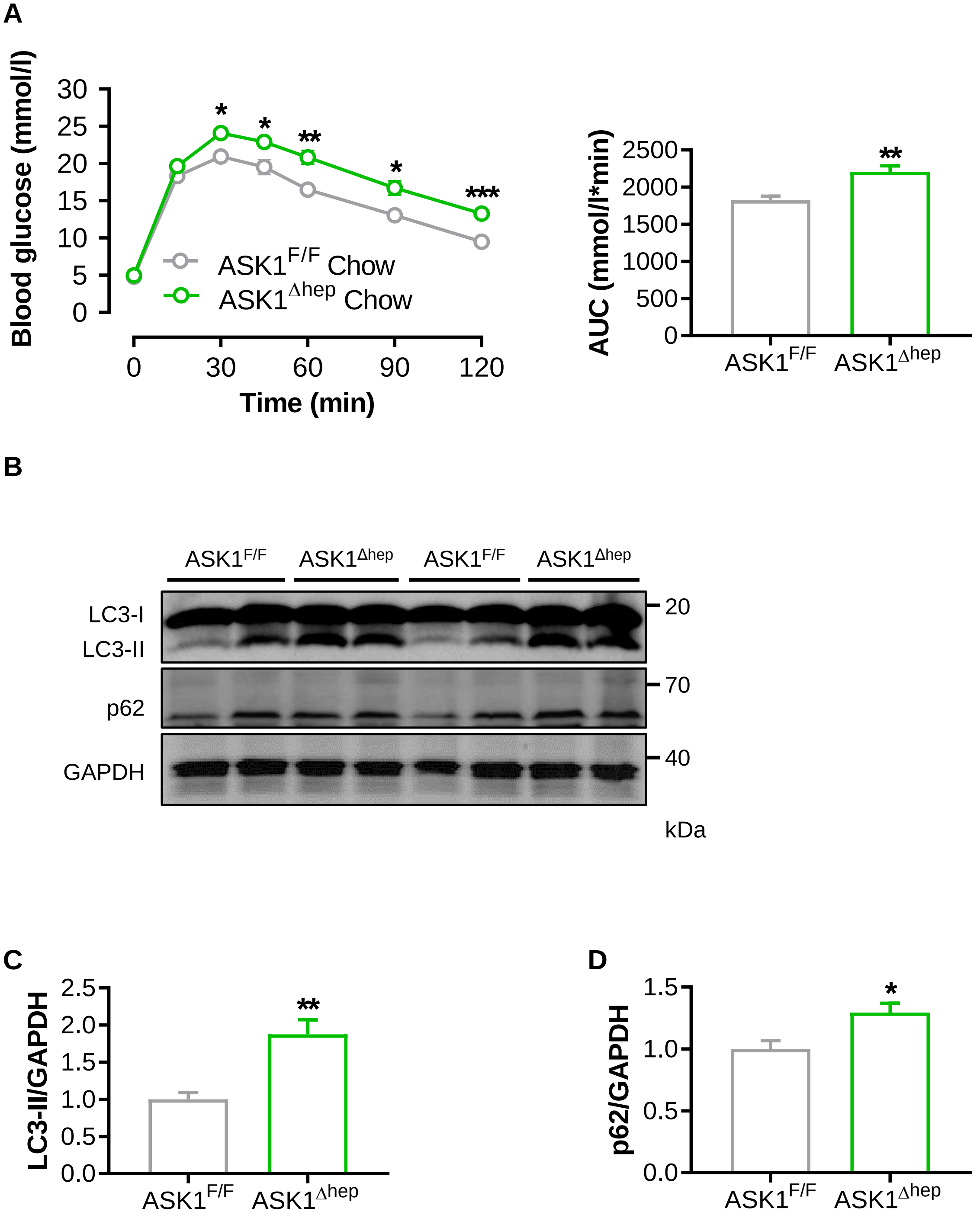

Reduced autophagy in 15 month-old hepatocyte-specific ASK1 knockout mice

(A) Intraperitoneal (i.p) glucose-tolerance test and corresponding AUC in mice fed a chow diet for 15 months ($ASK1^{F/F}$ n=6, ○; $ASK1^{\Delta hep}$ n=8, ○). Shown is one representative Western blot from two independent experiments (B) and quantification of protein levels (C-D) of respective proteins in livers of 15 month-old chow-fed mice ($ASK1^{F/F}$ n=7; $ASK1^{\Delta hep}$ n=7). All values are expressed as mean ± SEM. *p<0.05; ***p<0.001. Statistical test used: *t*-test.

Appendix Figure 4

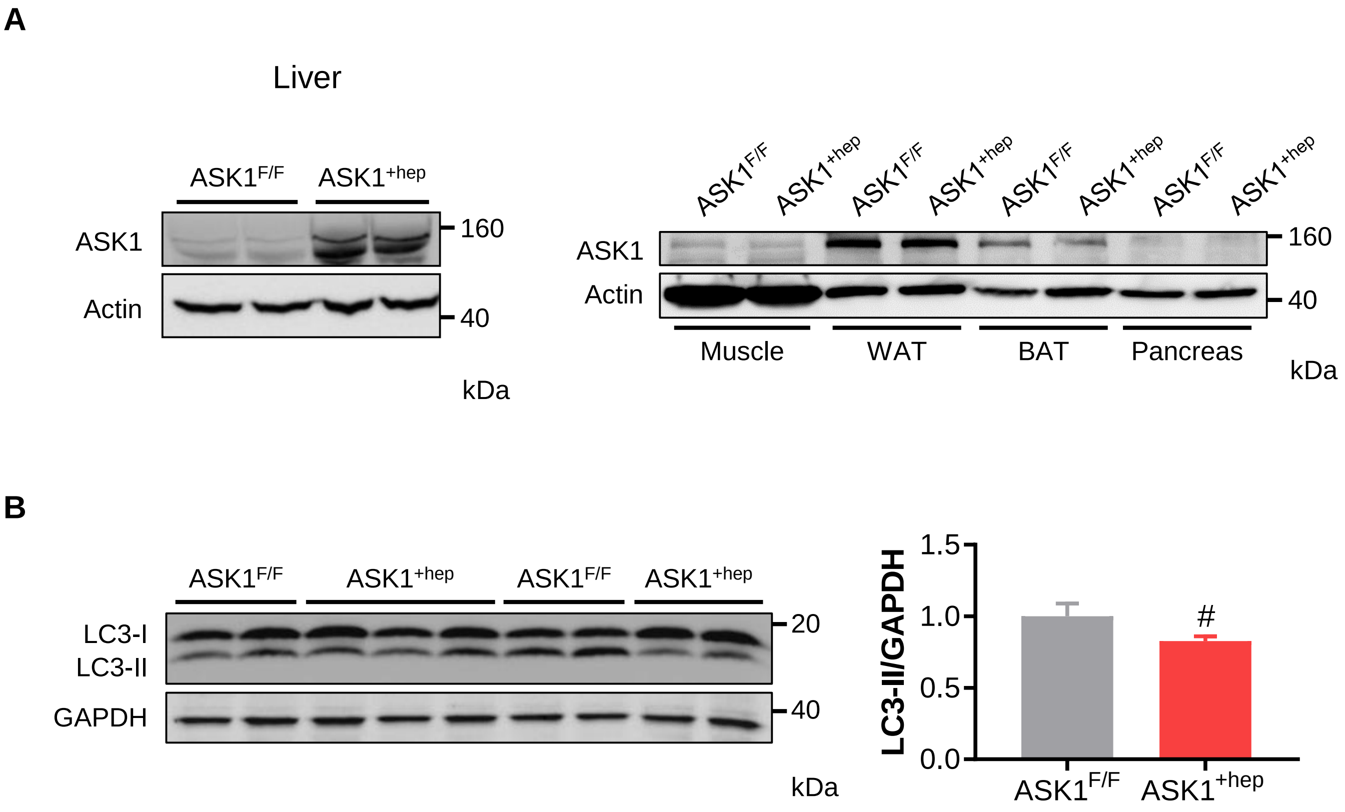

Liver-specific ASK1 overexpressing mice

(A) Protein levels of ASK1 in lysate of livers harvested from Rosa26-ASK1$^{F/F}$ and Rosa26-ASK1^{+hep} mice. (B) Shown is one representative Western blot from two independent experiments and quantification of protein levels of LC3-II in livers of HFD-fed mice (ASK1$^{F/F}$ n=8; ASK1^{+hep} n=9). All values are expressed as mean ± SEM. $^{\#}p<0.1$. Statistical test used: *t*-test.

Appendix Figure 5

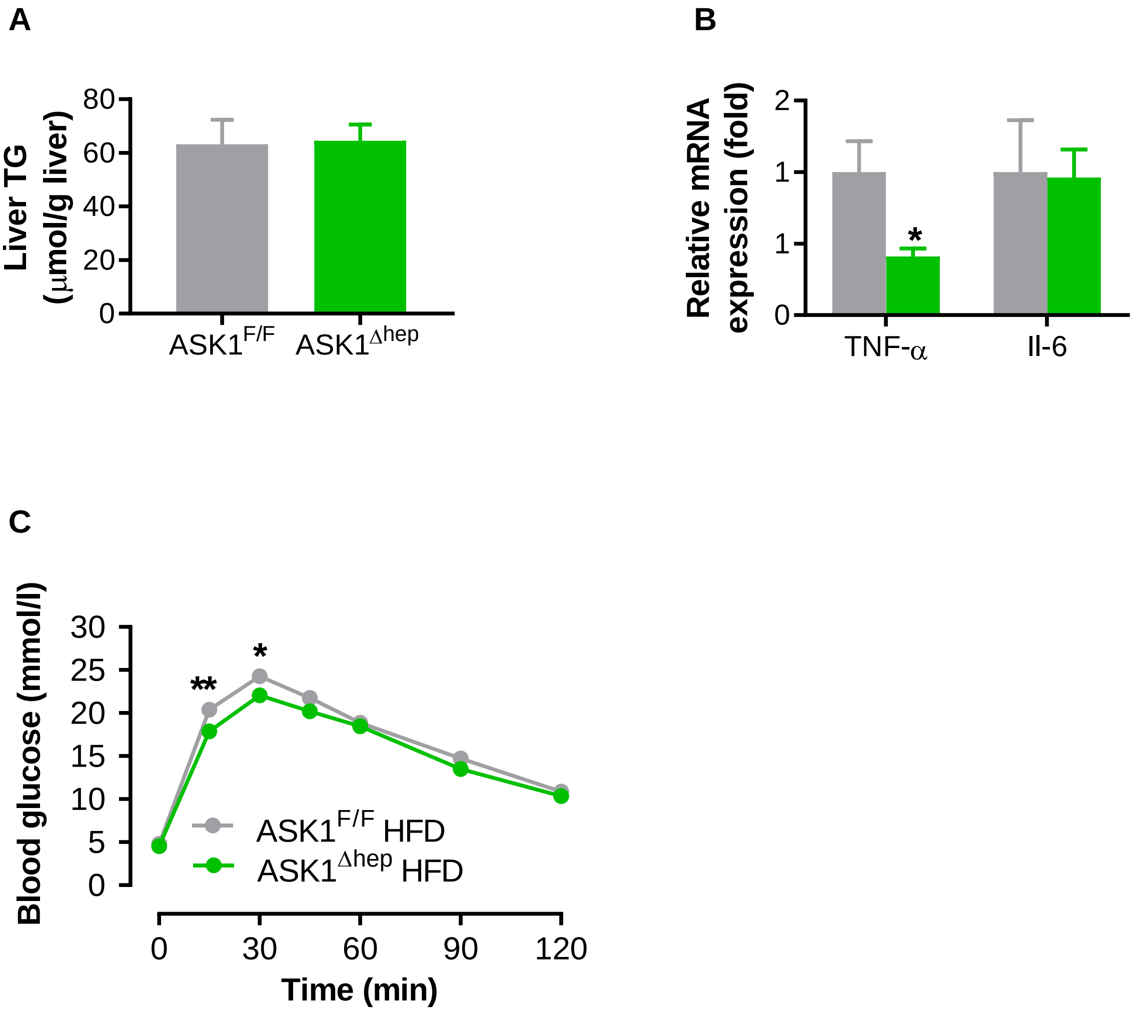

Improved glucose tolerance in hepatocyte-specific ASK1 knockout mice after 6 weeks of HFD

(**A**) Liver triglyceride (TG) content ($ASK1^{F/F}$ n=5; $ASK1^{\Delta hep}$ n=6) after 6 weeks of HFD. (**B**) Relative mRNA expression of *Tnf-α* ($ASK1^{F/F}$ n=4; $ASK1^{\Delta hep}$ n=5) and *Il-6* ($ASK1^{F/F}$ n=5; $ASK1^{\Delta hep}$ n=6) in liver after 6 weeks of HFD. (**C**) Intraperitoneal (i.p) glucose-tolerance test ($ASK1^{F/F}$ n=17; $ASK1^{\Delta hep}$ n=16) after 6 weeks of HFD. All values are expressed as mean ± SEM. *p<0.05; **p<0.01. Statistical test used: *t*-test.

Appendix Table 1 Clinical characteristics of human subjects

	Lean (n=14)	Overweight/Obese (n=42)
Age (years)	62 ± 12	70 ± 12
Men/women (n)	10/4	20/22
Type 2 diabetes (n)	3	20
Body mass index (kg/m^2)	24.5 ± 0.2	34.7 ± 0.9***
Body fat (%)	23.6 ± 0.9	36.6 ± 1.4***
Fasting plasma glucose (mmol/l)	5.6 ± 0.2	5.8 ± 0.1
Fasting plasma insulin (pmol/l)	60 ± 25	21 ± 22***
HbA1c (%)	5.5 ± 0.1	5.8 ± 0.1[#]
Total cholesterol (mmol/l)	4.5 ± 0.2	5.5 ± 0.1***
LDL-cholesterol (mmol/l)	2.9 ± 0.2	3.2 ± 0.1
HDL-cholesterol (mmol/l)	1.4 ± 0.1	1.3 ± 0.1
Triglycerides (mmol/l)	1.2 ± 0.2	2.1 ± 0.1***
Free fatty acids (mmol/l)	0.46 ± 0.07	0.64 ± 0.05

Values are expressed as mean ± SEM. [#]p=0.06, ***p<0.001. Statistical test used: *t*-test.

Appendix Table 2 Plasma metabolic analysis of chow and HFD-fed ASK1$^{F/F}$ and ASK1$^{\Delta hep}$ mice

	Chow diet		HFD	
	ASK1$^{F/F}$ (n=5-8)	ASK1$^{\Delta hep}$ (n=8)	ASK1$^{F/F}$ (n=6-9)	ASK1$^{\Delta hep}$ (n=7-14)
Glucose (mmol/l)	6.8 ± 0.7	6.9 ± 0.8	11.6 ± 1.6	12.3 ± 1.1
Insulin (ng/ml)	0.9 ± 0.3	0.5 ± 0.1	1.0 ± 0.2	1.5 ± 0.2
FFA (mmol/l)	1.9 ± 0.1	1.7 ± 0.1	1.5 ± 0.1	1.4 ± 0.1
Glycerol (µmol/l)	542 ± 31	580 ± 80	668 ± 59	805 ± 78
TG (mg/dl)	27.3 ± 2.0	28.0 ± 2.0	24.8 ± 2.2	27 ± 3.4
TC (mg/dl)	37.7 ± 3.6	38.4 ± 2.8	67.2 ± 5.3	73.4 ± 5.4
ALT (U/l)	31 ± 2	40 ± 3*	55 ± 9	91 ± 11*
AST (U/l)	120 ± 17	150 ± 28	182 ± 24	234 ± 37
IL-6 (pg/ml)	17.0 ± 3.7	23.0 ± 4.2	22.2 ± 3.9	12.6 ± 1.3
TNF-α (pg/ml)	10.0 ± 1.0	11.2 ± 1.0	10.4 ± 0.8	12.9 ± 1.1
IL-10 (pg/ml)	13.9 ± 3.0	16.4 ± 3.3	20.8 ± 5.8	20.1 ± 1.5
KC (pg/ml)	55.5 ± 7.8	61.8 ± 7.8	69.4 ± 12.8	72.5 ± 9.2

Six weeks-old male mice were fed either a chow or a high fat diet (HFD) for 20 weeks and fasted overnight for blood sampling. Values are expressed as mean ± SEM. *$p < 0.05$ indicates significant difference between genotypes under respective diets. Statistical test used *t* test. FFA = non-esterified free fatty acid, TG = triglyceride, TC = total cholesterol, ALT = alanine transaminase, AST = aspartate transaminase, IL-6/10= interleukin 6/10**,** TNF-α = tumor necrosis factor-alpha, KC= keratinocyte chemoattractant.

Appendix Table 3 Plasma metabolic analysis of control or ASK1 inhibitor treated mice

	Control (n=7)	ASK1 inhibitor (n=8)
Glucose (mmol/l)	12.6 ± 1.4	15.9 ± 1.2
FFA (mmol/l)	1.1 ± 0.1	1.5 ± 0.1**
Glycerol (μmol/l)	551 ± 93	411 ± 21
TG (mg/dl)	20.2 ± 2.5	244 ± 38***
TC (mg/dl)	77.1 ± 8.5	84 ± 4

Six weeks old C57BL/6J mice were fed a HFD for 11 weeks and treated either with ASK1 inhibitor or control during the last 5 weeks of HFD feeding. Mice were fasted overnight for blood sampling. Values are expressed as mean ± SEM. **$p < 0.01$, ***$p<0.001$. Statistical test used *t* test. FFA = non-esterified free fatty acid, TG = triglyceride, TC = Total cholesterol.

Appendix Table 4 *p*-Values

Figure	*p*-Value
Fig. 1D	***p<0.001, ****p<0.0001
Fig. 2A	***p<0.001
Fig. 2C	*p<0.05, **p<0.01, ***p<0.001
Fig. 2D	p=0.026
Fig. 2F	**p<0.01
Fig. 2G	*p<0.05, **p<0.01, ***p<0.001
Fig. 3B	*p<0.05, ***p<0.001
Fig. 3C	p=0.036 (Plin), p=0.047 (Vldlr)
Fig. 3D	p=0.026 (Il-6), p=0.056 (F4/80), p= 0.042 (Mcp1)
Fig. 3E	p=0.032 (Col1A1), p=0.019 (Tgf1β)
Fig. 3G	p<0.0001
Fig. 3I	p=0.017
Fig. 3J	p=0.082
Fig. 3K	p=0.0002
Fig. 4A	p=0.0002 (pJNK), p=0.0001 (pBCN1), p=0.0014 (LC3II), p=0.0005 (ATG12), p=0.0007 (p62)
Fig. 5A	*p<0.05, **p<0.01, ***p<0.001
Fig. 6A	p=0.012 (pASK1), p=0.040 (pJNK), p=0.002 (pBCN1), p=0.042 (LC3II), p=0.003 (p62)
Fig. 6C	p=0.018
Fig. 7B	p=0.020
Fig. 7C	p=0.007 (pJNK), p=0.005 (pBCN1), p=0.007 (ATG12)
Fig. 7D	p<0.0001
Fig. 7E	p=0.048 (IL-1β), p=0.092 (Tnf-α), p=0.064 (Mcp1)
Fig. 7F	p=0.016 (Col1A1), p= 0.054 (Tgfβ)

p-Values for data presented in main figures. For ANOVA (Newman-Keuls post hoc test), no exact *p*-values were given by the applied software GraphPad Prism 8.
