# Supplementary figures and images for: Liver ASK1 protects from non‐alcoholic fatty liver disease and fibrosis
Source: EMBO Mol Med. 2019 Jun 6;11(10):e10124. doi: 10.15252/emmm.201810124 (PMC6783644; doi:10.15252/emmm.201810124)

## Slide 1
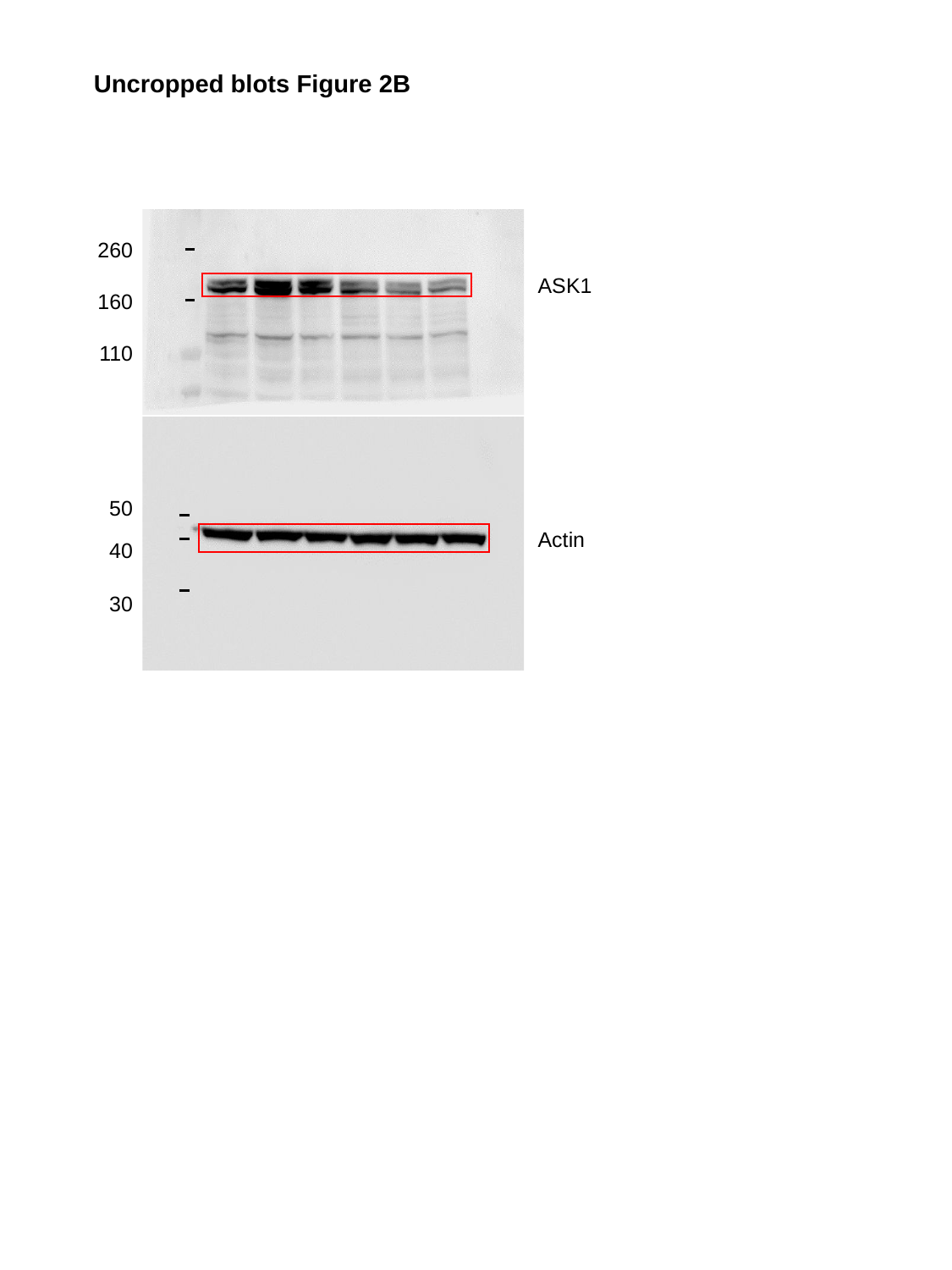

Uncropped blots Figure 2B
260
ASK1
160
110
50
Actin
40
30

## Slide 2
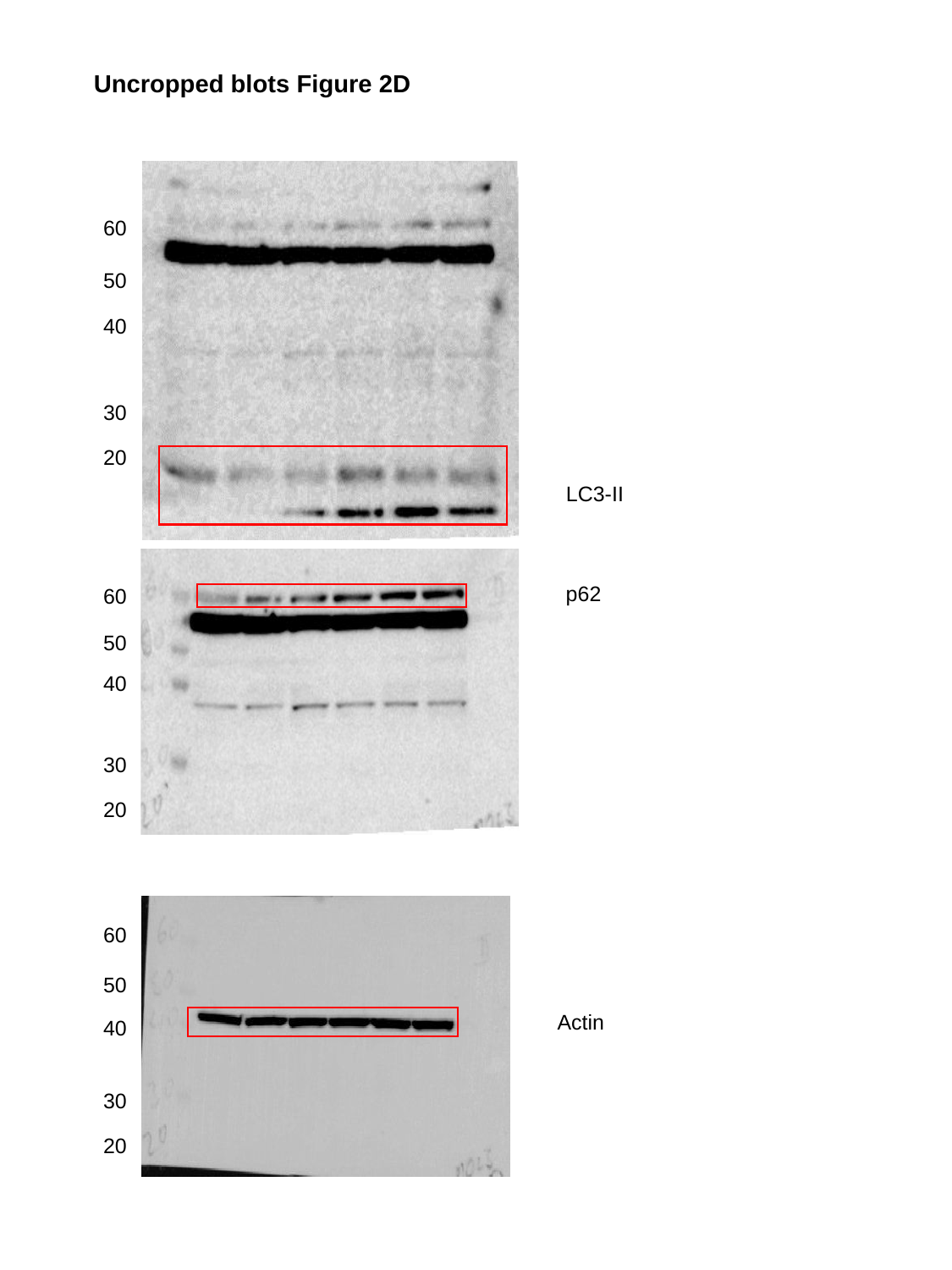

Uncropped blots Figure 2D
60
50
40
30
20
LC3-II
p62
60
50
40
30
20
60
50
Actin
40
30
20

Supplement: Supplementary file 3 — Source Data for Figure 2 [file EMMM-11-e10124-s002.pptx]

## Slide 1
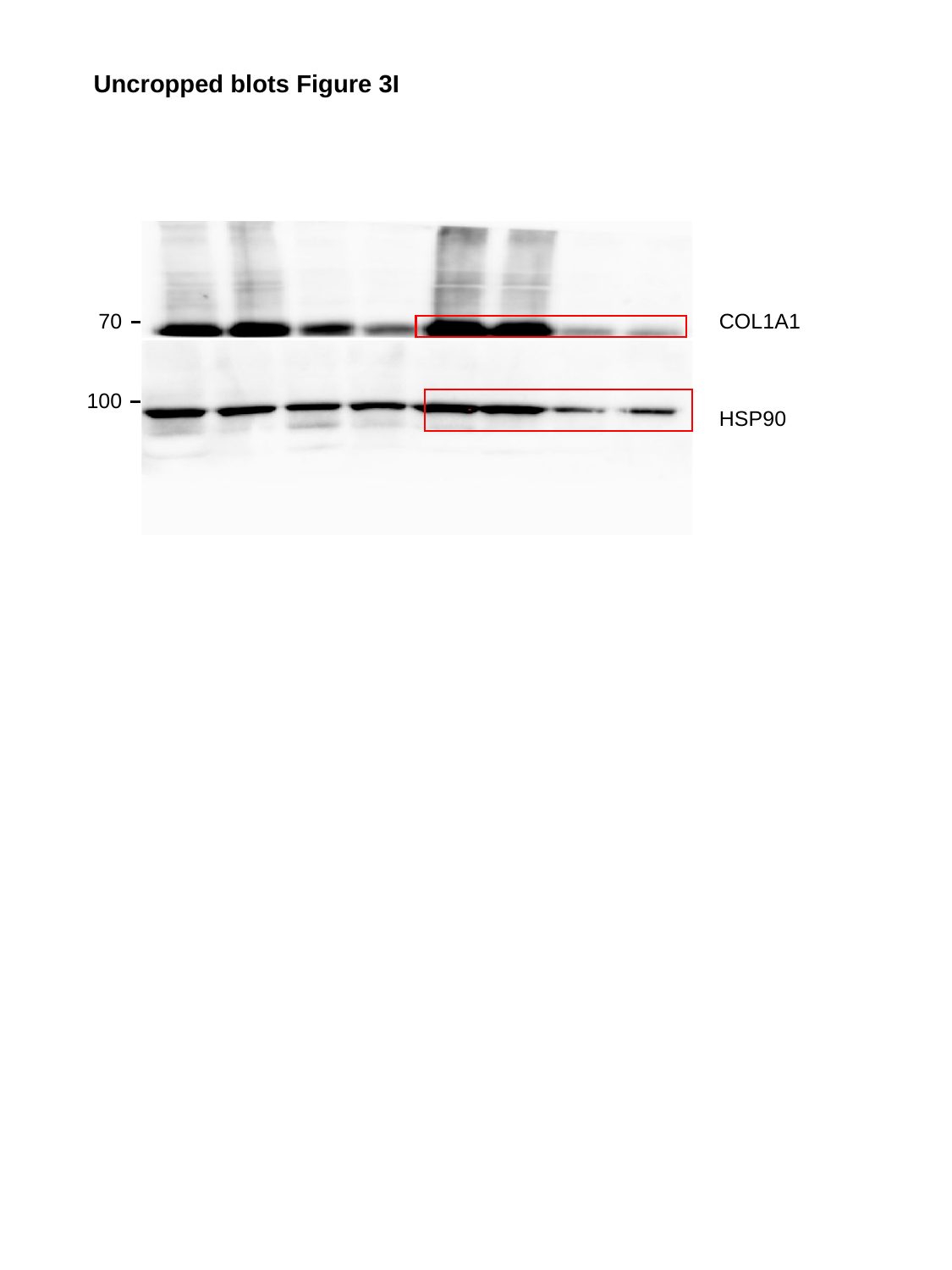

Uncropped blots Figure 3I
70
COL1A1
100
HSP90

Supplement: Supplementary file 4 — Source Data for Figure 3 [file EMMM-11-e10124-s003.pptx]
