# Supplementary material for: Liver ASK1 protects from non‐alcoholic fatty liver disease and fibrosis
Source: EMBO Mol Med. 2019 Jun 6;11(10):e10124. doi: 10.15252/emmm.201810124 (PMC6783644; doi:10.15252/emmm.201810124)
Supplement: Supplementary file 5 — Source Data for Figure 4 [file EMMM-11-e10124-s004.pptx]

## Slide 1
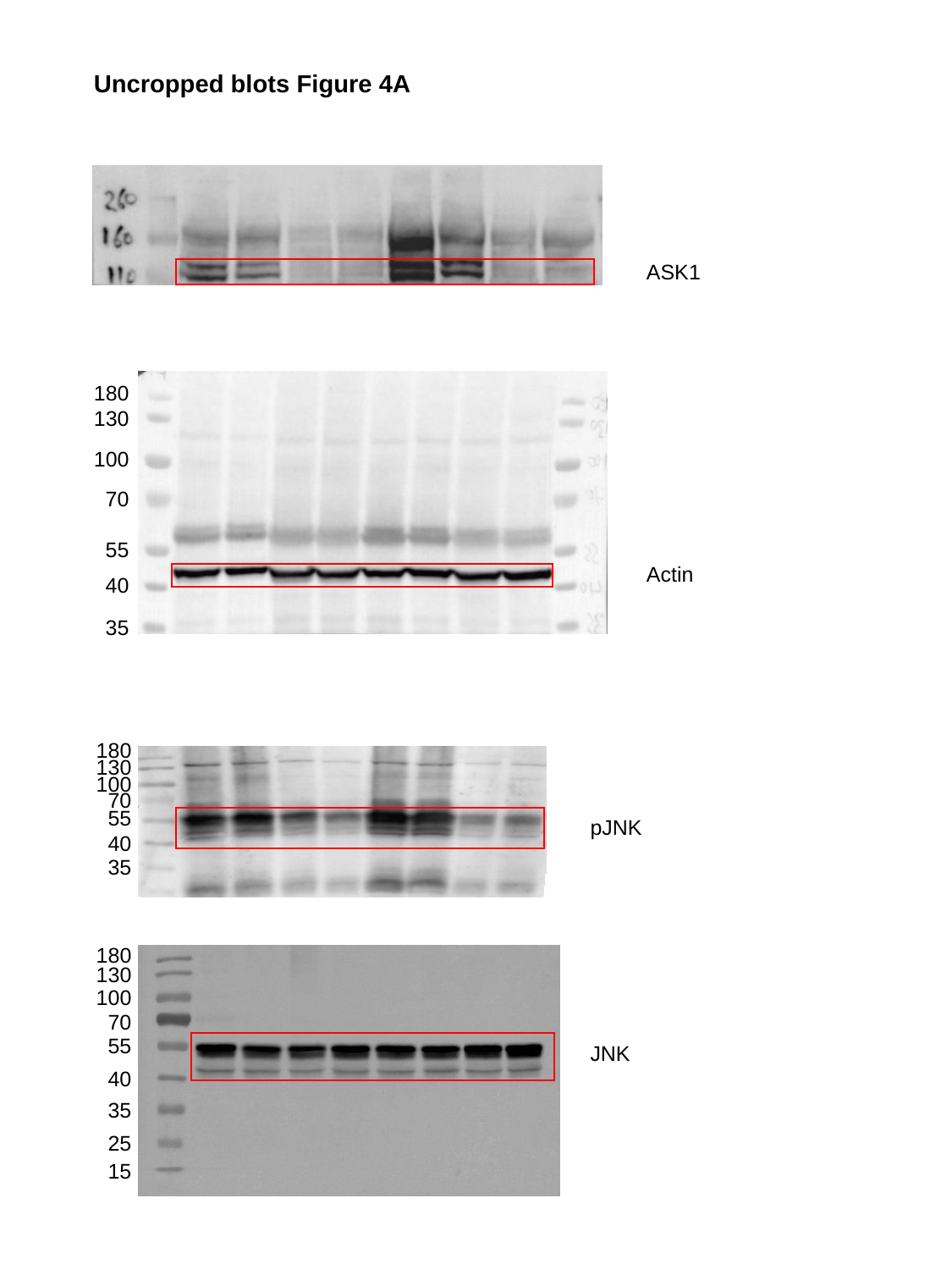

Uncropped blots Figure 4A
ASK1
180
130
100
70
55
Actin
40
35
180
130
100
70
55
pJNK
40
35
180
130
100
70
55
JNK
40
35
25
15

## Slide 2
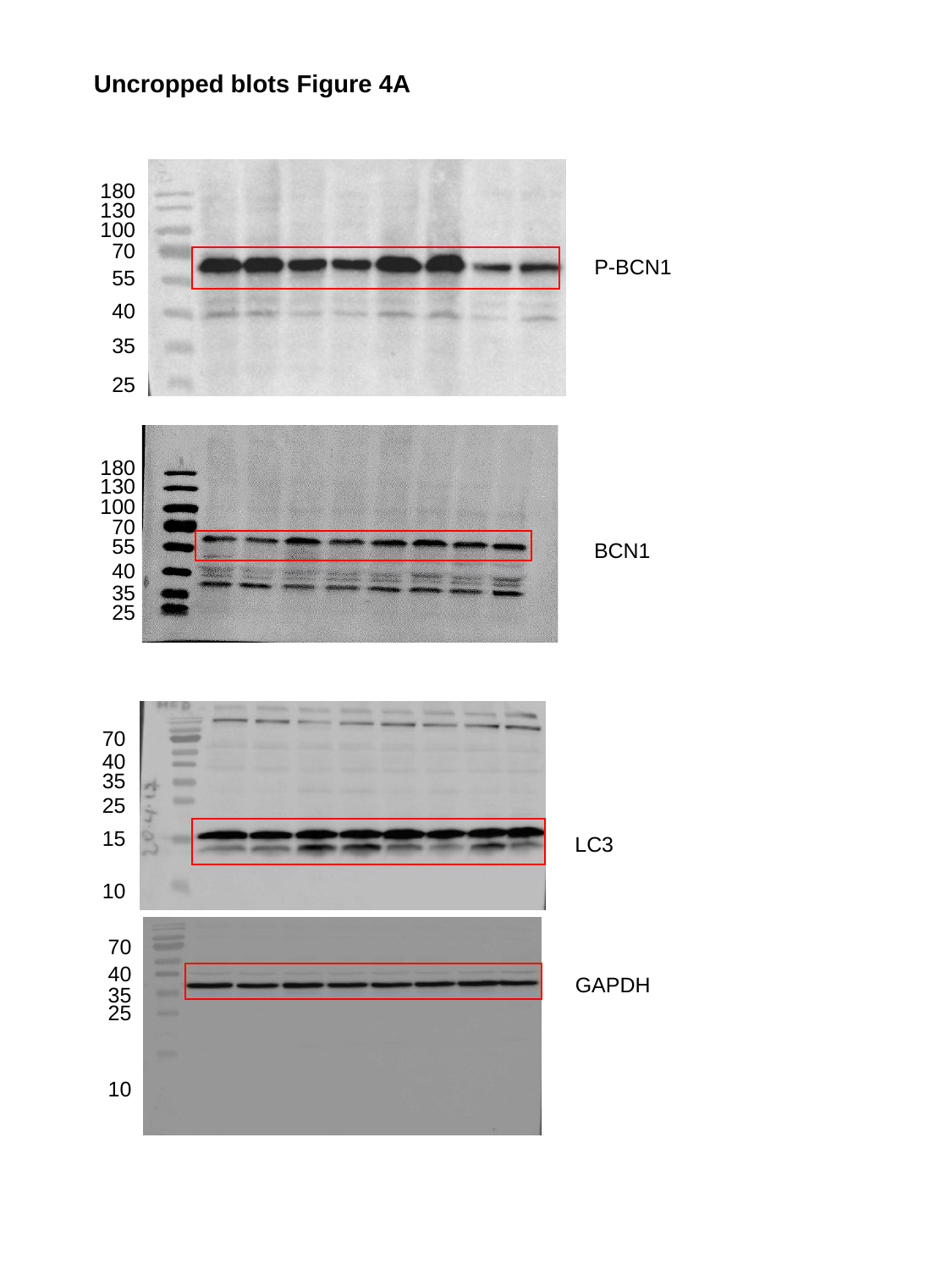

Uncropped blots Figure 4A
180
130
100
70
P-BCN1
55
40
35
25
180
130
100
70
55
BCN1
40
35
25
70
40
35
25
15
LC3
10
70
40
GAPDH
35
25
10

## Slide 3
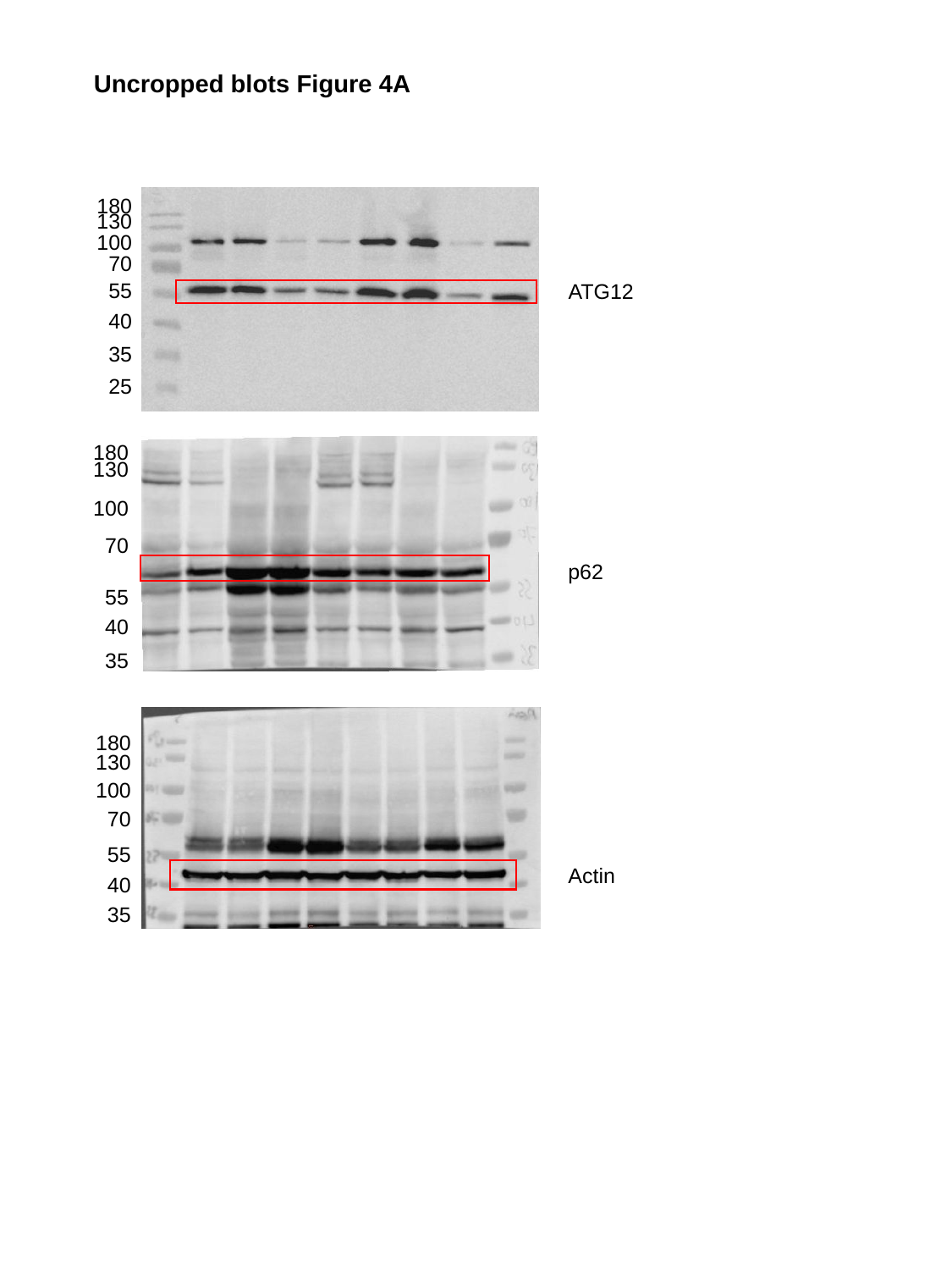

Uncropped blots Figure 4A
180
130
100
70
55
ATG12
40
35
25
180
130
100
70
p62
55
40
35
180
130
100
70
55
Actin
40
35
