# Supplementary material for: Liver ASK1 protects from non‐alcoholic fatty liver disease and fibrosis
Source: EMBO Mol Med. 2019 Jun 6;11(10):e10124. doi: 10.15252/emmm.201810124 (PMC6783644; doi:10.15252/emmm.201810124)
Supplement: Supplementary file 6 — Source Data for Figure 6 [file EMMM-11-e10124-s005.pptx]

## Slide 1
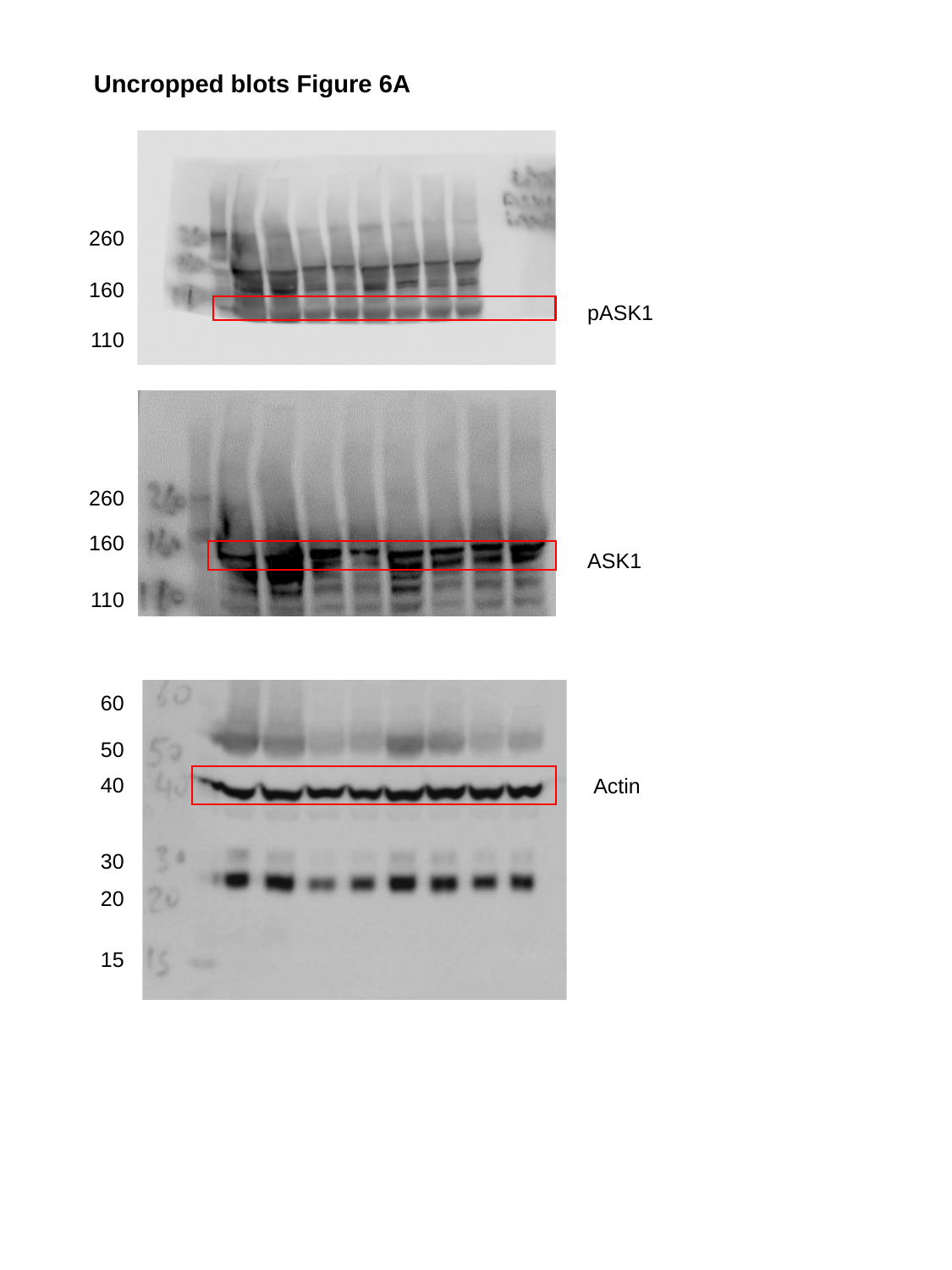

Uncropped blots Figure 6A
260
160
pASK1
110
260
160
ASK1
110
60
50
40
Actin
30
20
15

## Slide 2
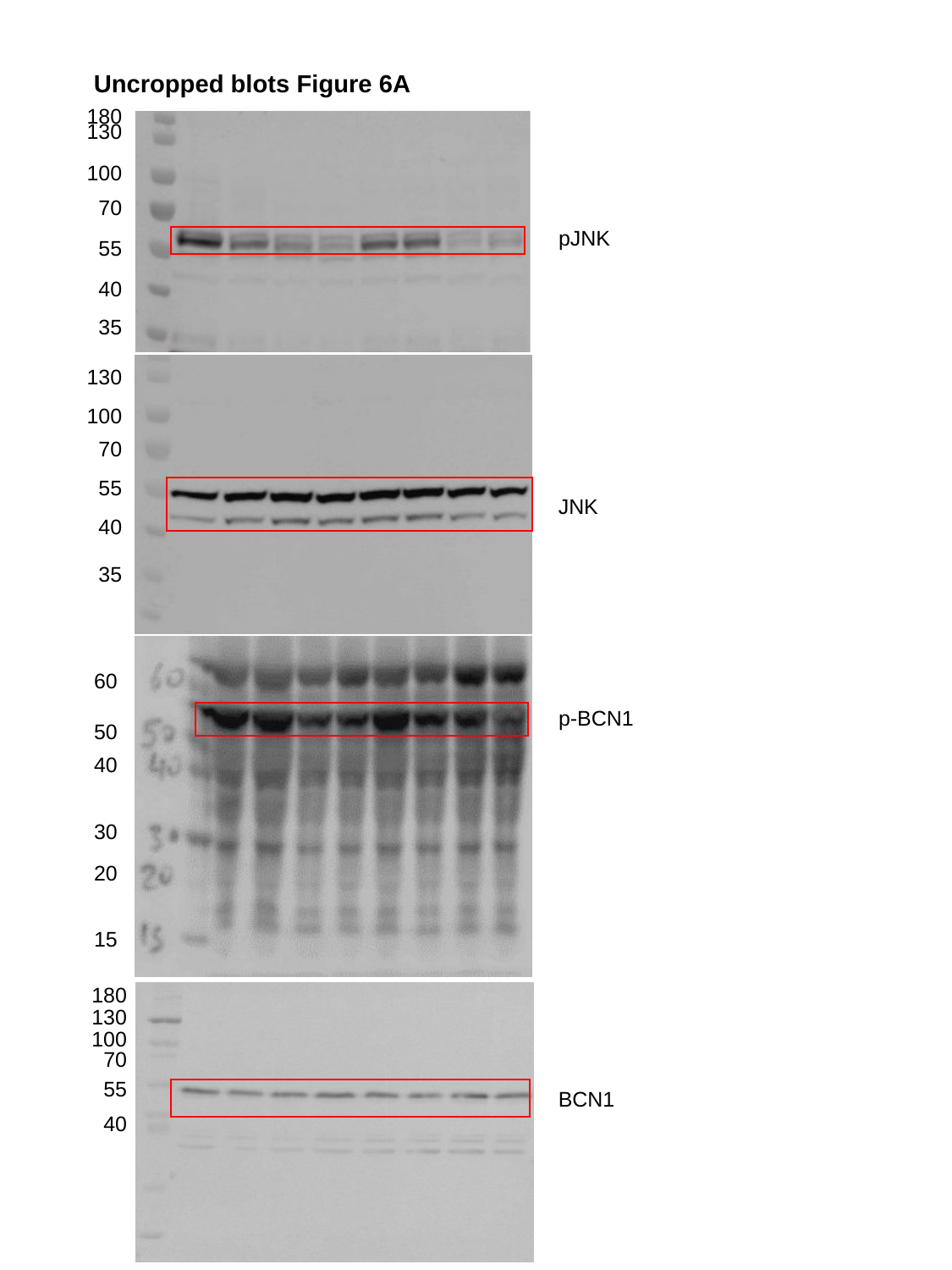

Uncropped blots Figure 6A
180
130
100
70
pJNK
55
40
35
130
100
70
55
JNK
40
35
60
p-BCN1
50
40
30
20
15
180
130
100
70
55
BCN1
40

## Slide 3
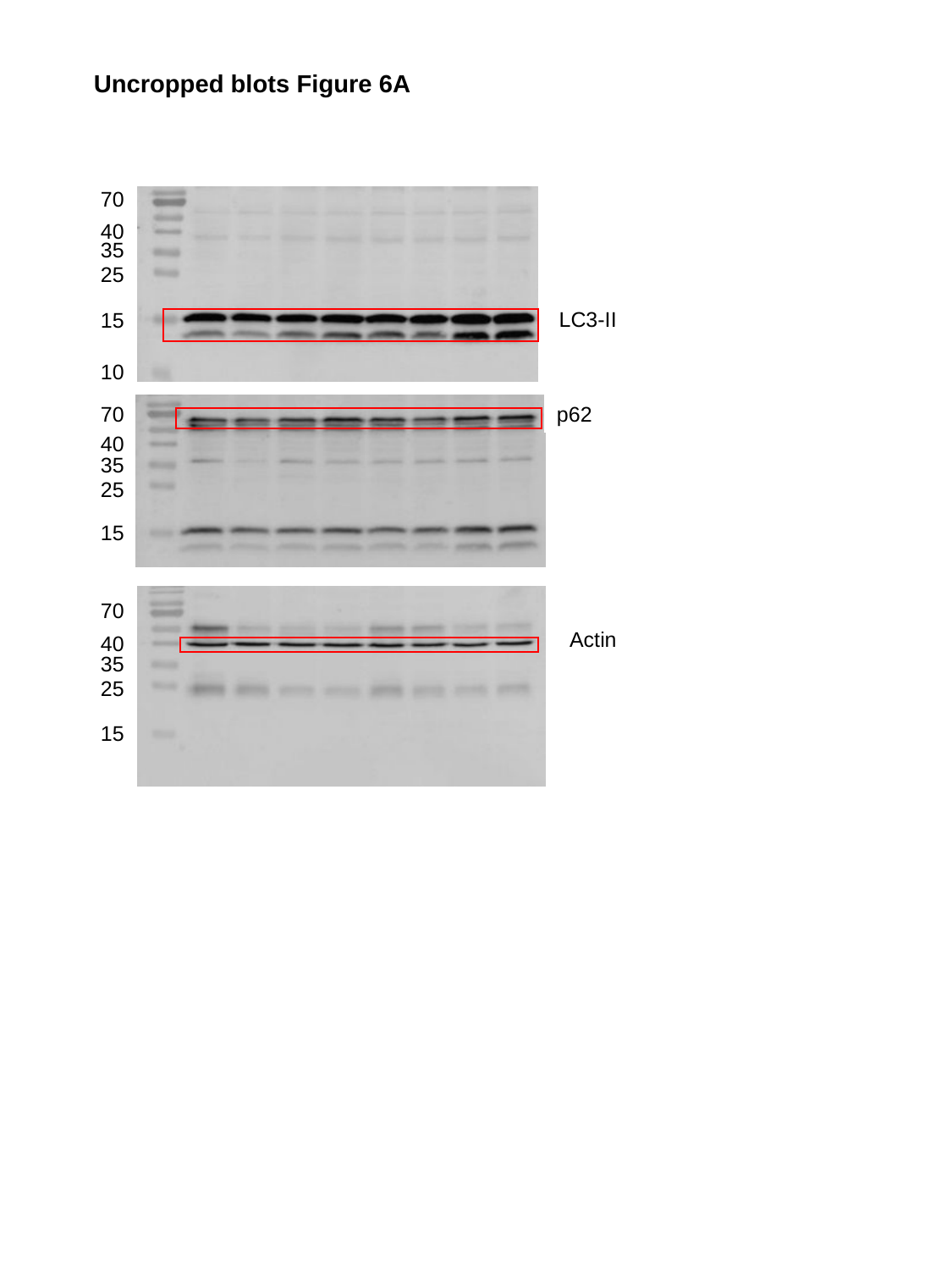

Uncropped blots Figure 6A
70
40
35
25
LC3-II
15
10
p62
70
40
35
25
15
70
Actin
40
35
25
15
