# Supplementary material for: Liver ASK1 protects from non‐alcoholic fatty liver disease and fibrosis
Source: EMBO Mol Med. 2019 Jun 6;11(10):e10124. doi: 10.15252/emmm.201810124 (PMC6783644; doi:10.15252/emmm.201810124)
Supplement: Supplementary file 7 — Source Data for Figure 7 [file EMMM-11-e10124-s006.pptx]

## Slide 1
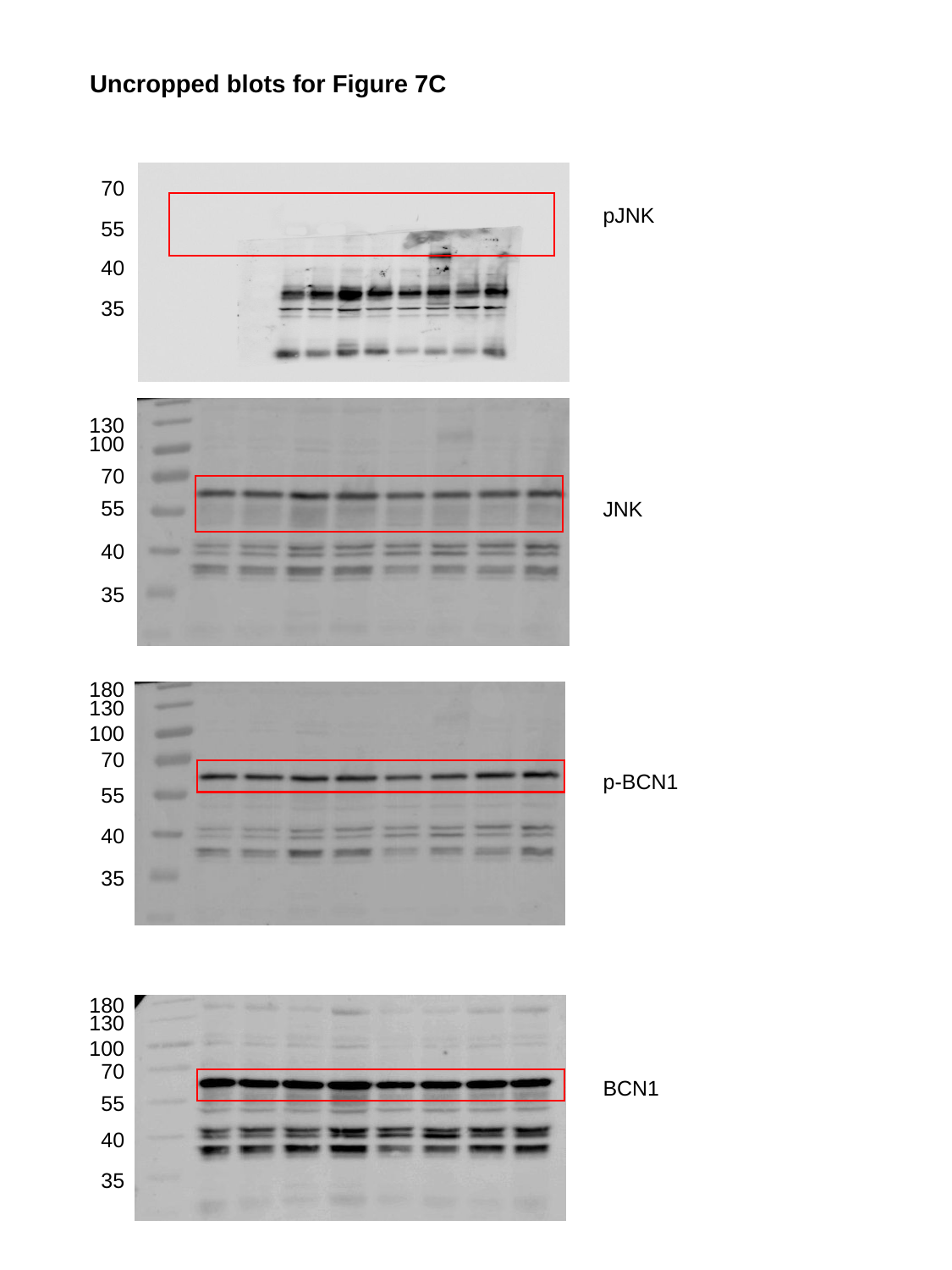

Uncropped blots for Figure 7C
70
pJNK
55
40
35
130
100
70
55
JNK
40
35
180
130
100
70
p-BCN1
55
40
35
180
130
100
70
BCN1
55
40
35

## Slide 2
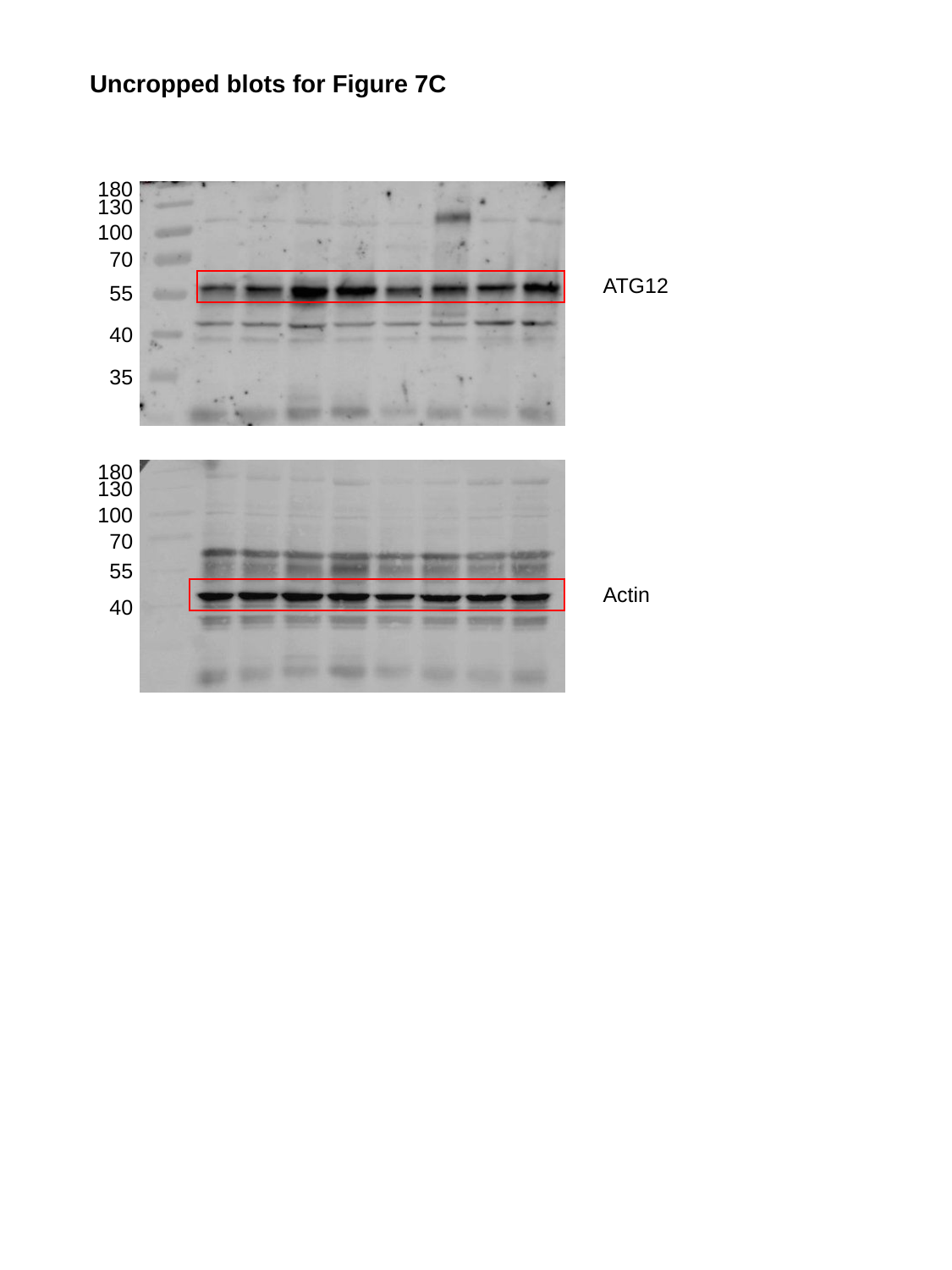

Uncropped blots for Figure 7C
180
130
100
70
ATG12
55
40
35
180
130
100
70
55
Actin
40
